# WGTMM: WGAN with Transformer Feature Matching for Generating fMRI Data in MCI Patients

**DOI:** 10.3390/brainsci16070665

**Published:** 2026-06-25

**Authors:** Bocheng Wang

**Affiliations:** School of Media Engineering, Communication University of Zhejiang, No.998, Xueyuan Street, Hangzhou 310018, China; wangboc@cuz.edu.cn

**Keywords:** Alzheimer’s disease, GAN, Transformer, ViT, feature matching

## Abstract

**Highlights:**

**What are the main findings?**

- WGTMM integrates feature matching and Wasserstein GAN to directly generate fMRI BOLD time-series data from pink noise across four cognitive stages.
- The generated fMRI data achieved lower KL divergence and improved multi-class classification performance compared with conventional GAN-based methods.

**What are the implications of the main findings?**

- The proposed framework provides an effective data augmentation strategy for limited Alzheimer’s disease fMRI datasets.
- WGTMM combined with VTFF analysis revealed progressive cortical alterations associated with cognitive decline and disease progression.

**Abstract:**

Background: The emergence of generative adversarial networks has laid the groundwork for data augmentation, addressing challenges of missing training data in various research scenarios. However, simulating functional magnetic resonance imaging (fMRI) data remains particularly challenging, especially for populations with varying degrees of mild cognitive impairment (MCI). Effectively characterizing and capturing the mechanisms of brain function variations poses a critical issue in cognitive neuroscience. This study aims to simulate and analyze synthetic fMRI blood-oxygen-level-dependent (BOLD) signals across four cognitive stages: healthy control (HC), early MCI (EMCI), late MCI (LMCI), and Alzheimer’s disease (AD). Methods: We propose WGTMM, an innovative method that integrates the Vision Transformer for fMRI (VTFF) into a generative adversarial network architecture. Crucially, WGTMM directly generates fMRI time-series data from pink noise rather than modeling in a latent space, thereby preserving rich temporal dynamics. The framework incorporates a Wasserstein GAN (WGAN) with feature matching to enhance generation quality and mitigate mode collapse. Results: demonstrate that WGTMM-generated fMRI data exhibit lower Kullback-Leibler (KL) divergence compared to traditional GAN and WGAN models, indicating a closer resemblance to real datasets from the Alzheimer’s Disease Neuroimaging Initiative (ADNI). Furthermore, when applied to data augmentation, the synthetic data substantially improve multi-class classification performance. Conclusions: WGTMM not only enriches training datasets but also provides new insights into spatial biomarkers of cognitive decline. By leveraging VTFF to investigate class token attention patterns across 360 brain regions, this study reveals monotonic weight variations along disease stages in key cortical areas, including the rostral Area 6, the primary sensory cortex, and PFm near Wernicke’s area, offering a fine-grained exploration of disease progression.

## 1. Introduction

Cognitive dysfunction in the brain is a progressive neurodegenerative disease characterized by a decline in various abilities, including comprehension, cognition, memory, organization, coordination, and auditory-visual processing. This condition places a significant burden on both the affected individuals and society, making it one of the most serious neurological disorders worldwide. Currently, various hypotheses regarding the cause of this disease have emerged, including abnormal gene expression, deposition of β-amyloid plaques, and alterations in brain neural tissue; however, a definitive explanation remains elusive, necessitating further in-depth research. With the aid of rapidly advancing neuroimaging technologies, researchers can visually and accurately characterize the structural and functional features of the brain. This approach helps to elucidate the mechanisms underlying human brain function and facilitates the early identification and management of potential pathological risks associated with cognitive disorders. Ultimately, these efforts aim to inhibit the progression of the disease. Numerous studies have shown that early prediction based on brain morphology and functional patterns can be effectively conducted by integrating artificial intelligence techniques with neuroimaging data analysis [1,2,3]. The advantages of artificial intelligence primarily include two aspects: first, they enable the training of models for the early identification of cognitive impairment; second, they serve as a feature selection tool to analyze the patterns of brain variations. Jin et al. [4] combined structural magnetic resonance imaging (sMRI) data with resting-state functional MRI (rs-fMRI) data, utilizing cortical thickness, brain structural network features, and functional brain network characteristics across different frequency bands from 104 sets of Alzheimer’s Disease Neuroimaging Initiative (ADNI) data as inputs for machine learning analysis. They compared several classical algorithms, including support vector machines (SVM), random forests, and K-nearest neighbors, ultimately proposing a method termed RSFS that achieved a classification model with an accuracy of 89.80%. Grueso et al. [5] conducted a quantitative analysis and literature review of 116 studies on Alzheimer’s disease methodologies. They found that most cognitive impairment research utilized MRI and PET imaging techniques, with sample data primarily sourced from the ADNI. Moreover, the most frequently used artificial intelligence algorithms were SVM, employed in 75.4% of studies, and convolutional neural networks (CNN), used in 78.5% of studies. It can be concluded that traditional artificial intelligence methods have made significant progress in identifying cognitive impairments, particularly in binary classification problems. Additionally, the understanding of how variations in brain connectivity influence cognitive disorders has gradually gained widespread acceptance. However, in practice, these models often encounter situations involving more than two classes of samples. In previous research, we explored methods for multiclass cognitive impairment recognition [6,7], proposing models such as the monotonic progressive change hypothesis. Nonetheless, achieving precise classification of three or more types of brain diseases remains a significant challenge that requires urgent attention.

Compared to three-dimensional brain structural imaging, functional magnetic resonance data provide rich time-varying information, specifically in the form of time series that reflect brain blood oxygen level-dependent (BOLD) signals. For processing these time series, a deep learning technique known as the Transformer has emerged in recent years, alongside traditional machine learning methods [8]. This technology has been extensively studied across various domains, achieving significant advancements in areas such as long text translation, speech recognition, image classification, and video processing [9]. In neuroimaging research, some researchers have applied the Transformer architecture to the processing of fMRI data [10,11], with the goal of preserving the intrinsic relationships of BOLD signals across various brain regions at different time points in long-distance time series. This type of associative information is often regarded as one of the most important essential mechanisms in pattern recognition. Sarraf et al. [12] proposed an optimized visual Transformer architecture known as OViTAD, which utilizes both structural and functional magnetic resonance data to conduct predictive analyses at different stages of Alzheimer’s disease. Hu et al. [13] proposed a method that combines classical Visual Geometry Group (VGG) architecture with Transformer, employing a sliding window modeling approach on longitudinal data from patients with MCI. This method utilizes a temporal attention mechanism to establish patterns of brain structural changes associated with disease progression.

Although Transformers have achieved remarkable success across various fields, their fundamental advantage lies in the vast amounts of multimodal multimedia data, such as text, audio, images, and video readily available on the Internet. In cognitive neuroscience research, the development of Transformers remains significantly limited, primarily due to the substantial gap in data scale between neuroimaging and other fields. Therefore, maximizing the acquisition of neuroimaging data specific to brain disorders or improving model performance through data augmentation techniques remains a central challenge in cognitive neuroscience research. Early fMRI data augmentation methods primarily involved traditional techniques such as image rotation, motion correction, artifact removal, and the addition of artificial noise. With technological advances, generative models have gradually been introduced to synthesize realistic fMRI time series. For example, Nguyen et al. [14] proposed a co-registration-based preprocessing method grounded in anatomical knowledge to generate fMRI images that preserve authentic brain morphological features. Qiang et al. [15] developed a Deep Recurrent Variational Autoencoder (DRVAE), leveraging the encoder of a Variational Autoencoder (VAE) to extract generalized temporal features from the assumed Gaussian latent space of the input data, and using the decoder to generate new samples to augment training datasets. These approaches provide effective solutions to mitigate the scarcity of neuroimaging data and contribute to enhancing classification performance by expanding the training sample pool. Among generative models, Generative Adversarial Networks (GANs) [16], which have significantly advanced image synthesis, have increasingly attracted attention for neuroimaging data augmentation.

GANs consist of a generator and a discriminator. The generator is responsible for producing synthetic data from noise to deceive the discriminator, while the discriminator focuses on distinguishing between real and synthetic data. Through multiple iterations of this adversarial process, both the generator and the discriminator progressively enhance their capabilities. In neuroimaging research, GANs can be employed to generate synthetic brain images to address the scarcity of real data. By analyzing the shared characteristics between real images and synthetic data, researchers can explore brain functioning patterns that are challenging to analyze intuitively in cognitive neuroscience. Zhang et al. [17] proposed a BSGAN-ADD research method that combines GAN-based brain slice image enhancement techniques with deep convolutional neural networks to extract higher-level brain features, achieving advanced classification and recognition of AD. Park et al. [18] proposed a novel conditional GAN network designed to synthesize high-quality 3D MRI images of patients at various stages of AD. However, while GANs generate augmented data, a significant challenge that has consistently troubled researchers is the phenomenon of mode collapse. This issue manifests in the quality of the generated data, as it leads to the repetitive creation of images with similar patterns during iterations, which is clearly detrimental to the enhancement of both the generator’s and the discriminator’s capabilities. Consequently, various techniques, such as Wasserstein GAN (WGAN) and Feature Matching [19,20], have emerged to address mode collapse and improve the quality of synthetic data.

This study leverages GAN technology by incorporating attention mechanisms into both the generator and the discriminator. The Vision Transformer (ViT) model [21], suitable for image classification, is utilized as a core component of the GAN, with the Transformer serving as a feature matching layer. Importantly, the proposed framework is designed to learn a constrained representation of the empirical data distribution within observed cohorts, rather than to generate novel biological brain states or model unseen disease trajectories. Synthetic samples are used only for distributional augmentation under controlled conditions.

## 2. Method

**Figure 1:**
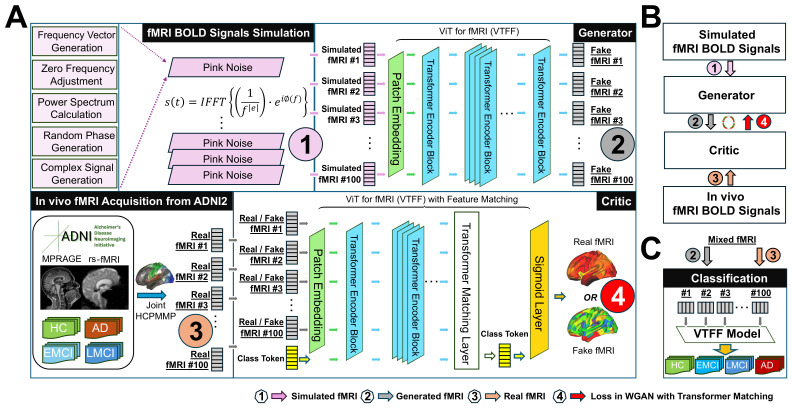
Proposed Wasserstein GAN network with Transformer feature matching (WGTMM) for adversarial generation of fMRI signals in patients with varying degrees of cognitive impairment. (**A**) In WGTMM architecture, ① Output of Pink Noise simulating the fMRI BOLD signal; ② Fabricated fMRI generated by the Generator; ③ Real fMRI data obtained from the ADNI database; ④ Output of the Critic network. (**B**) The adversarial training process between the Generator and the Critic. (**C**) VTFF-based classification module for downstream cognitive status prediction.

Figure 1 illustrates the overall framework and data analysis workflow of this study. The proposed method consists of five modules: the fMRI BOLD time series signal simulation module, the fMRI generator module, the fMRI critic module (discriminator), the real fMRI signal acquisition module (shown in Figure 1A), and the classifier module (shown in Figure 1C). Figure 1B depicts the workflow among the output components outlined in Figure 1A. These include: ① simulated fMRI BOLD signals, ② synthetic fMRI generated by the generator, ③ real fMRI data, and ④ the decision outcome produced by the critic. The green circular loop in the middle highlights the iterative training process between the generator and the critic.

The fMRI simulation module is designed to produce random data resembling resting-state fMRI signals, which is then input into the generator (the upper half of Figure 1A, Output ① in Figure 1B, indicated by the pink arrow). The generator uses a specific neural network architecture to create fabricated fMRI data (shown on the right side of Figure 1). This fabricated fMRI signal (Output ② in Figure 1B, indicated by the gray arrow) is then input alongside the real fMRI signal (Output ③ in Figure 1B, indicated by the orange arrow) into the critic. The critic assesses the authenticity of the input data and feeds the results back to the generator (Output ④ in Figure 1B, indicated by the red arrow). This iterative process continues until a balance is reached between the generator and the critic, after which the generator’s fabricated results are mixed with real fMRI data (in the classifier module of Figure 1C). This mixture is used to train the classifier model, examining whether it benefits from the adversarially generated fMRI data compared to a model trained solely on real fMRI signals. This approach aims to enhance the model’s ability to identify patients with varying degrees of cognitive impairment and helps analyze the commonalities in fMRI modalities between real and generated cognitive impairment patient brain neuroimaging.

During implementation, we utilized the PyTorch 2.5.1 framework, accessed on 1 October 2025 (https://pytorch.org/) for model training, with the following hardware specifications: i9-13980HX 2.2 GHz CPU, 32 GB RAM, and NVIDIA GeForce RTX 4080 Laptop GPU.

### 2.1. RS-fMRI BOLD Signals Simulation

In the upper left corner of Figure 1, the method for simulating fMRI BOLD time series is illustrated. To more effectively guide the neural network in generating realistic fMRI signals from patients with varying degrees of cognitive impairment, the study employs random pink noise [22] as a simulation for resting-state fMRI, instead of using completely random noise as input to the generator, as seen in other GAN networks. This approach mimics the spontaneous fluctuations of low-frequency BOLD signals. Unlike the simulation of task-based fMRI signals, which requires additional convolution of the hemodynamic response function (HRF) to establish a link between external stimuli and blood oxygen level signals, this method directly employs pink noise—characterized by its spectral energy density increasing with decreasing frequency—as a representative of resting-state fMRI. The mathematical process is outlined in (1) [23].

(1) $$s\left(t\right)=IFFT\left\{\left(\frac{1}{f^{\left|e\right|}}\right)\cdot e^{i\Phi (f)}\right\}$$

In this equation, IFFT denotes the inverse Fourier transform, *f* represents frequency, *e* is the exponent, and Φ(f) is the random phase uniformly distributed in the interval [0,2π]. First, a frequency array ranging from 0 to half the sampling rate is generated (as illustrated in the Frequency Vector Generation Box), and the zero frequency direct current (DC) component is removed to avoid division by zero issues in $\frac{1}{f^{\left|e\right|}}$ (Zero Frequency Adjustment Step). Next, the power spectrum for these frequency components is calculated, and a random phase of the same length as the frequency array is generated. By applying the inverse fast Fourier transform, the time-domain signal s(t) is obtained (Complex Signal Generation Box), which serves as the simulation for resting-state fMRI (Pink Noise Box).

To ensure that this time-domain signal aligns with the real fMRI data, its length is defined to match that of the actual fMRI signals, both consisting of 100 time points. Additionally, the cortical parcellation method from Washington University Human Connectome Project Multi-Modal Parcellation (HCP MMP) [24,25] is utilized to align the fMRI data with sMRI, dividing the brain into 360 regions, with 180 regions in each hemisphere. Therefore, the total number of simulated fMRI signals in this study is 360, corresponding to the 360 brain regions, with each region containing 100 time points of fMRI simulation data. To facilitate batch training of these data in the neural network, the batch size is set to a commonly used value of 32, resulting in an output data shape of (32, 100, 360).

### 2.2. Generative Adversarial Network for fMRI Study

The GAN model originated in the field of computer image synthesis and has been widely applied in image enhancement to improve the performance of classification models [26]. In cognitive neuroscience, the application of GAN networks to process fMRI data to generate high-quality, realistic fMRI BOLD signals presents certain challenges [27]. First, the previously simulated fMRI random signal s(t) is used as the prior input to the GAN. This input is then fed into a learner $G(s\left(t\right);\theta_{G})$ with a specific neural network architecture, where $\theta_{G}$ represents the parameters of this neural network. The goal is to learn the distribution characteristics of real fMRI data and to produce sufficiently realistic fake fMRI.

Simultaneously, a discriminator is defined, which receives fMRI data from patients with varying degrees of cognitive impairment and the generated fake fMRI as inputs to the model $D(x;\theta_{D})$. Based on their labels (real or fake), the binary cross-entropy loss is computed using the BCELoss function. This loss function is then used to update the network parameters of *D*. The results of the discriminator’s judgments on the generated fMRI are compared to the true labels to update the network parameters of *G*, iterating through this adversarial process. During model training, the generator aims to create sufficiently realistic fMRI signals to deceive the discriminator, while the discriminator strives to distinguish between real and fake samples. Therefore, the overall loss function for the GAN is generally defined as in (2) [28]:

(2) $$\begin{array}{c} \min_{G}\max_{D}V\left(D,G\right)=E_{x∼p_{real}\left(x\right)}\left[\log D(x;\theta_{D})\right]+\\ E_{x∼p_{s(t)}\left(s\left(t\right)\right)}\left[\log\left(1-D(G\left(s\left(t\right);\theta_{G}\right);\theta_{D})\right)\right] \end{array}$$

In which, *G* aims to minimize the objective function V(D,G), while *D* seeks to maximize this objective function. The variable p represents the probability distribution of real or fake fMRI samples. The terms $E_{x∼p_{real}\left(x\right)}$ and $E_{x∼p_{s(t)}\left(s\left(t\right)\right)}$ denote the expected judgments for real fMRI and fake fMRI, respectively. The cross-entropy loss is computed using the logarithm function, which drives $E_{x∼p_{real}\left(x\right)}$ to yield outputs as close to 1 as possible for real fMRI, while $E_{x∼p_{s(t)}\left(s\left(t\right)\right)}$ aims to produce outputs as close to 0 as possible for fake fMRI.

### 2.3. Wasserstein Distance in GAN

Research [29] has found that fMRI GAN networks often exhibit instability during training, including difficulties in convergence and mode collapse. This is commonly observed in the field of image generation and is typically attributed to factors such as a lack of sample diversity and an overly powerful discriminator [30]. To enhance model performance, Wasserstein distance (3), also known as Earth-Mover distance, can be introduced as a replacement for the traditional cross-entropy loss function. This model was proposed by Martin et al. [31], who conducted a comparative analysis of four distance formulations, revealing the superior convergence properties of Wasserstein distance.

(3) $$W\left(p_{real},p_{s(t)}\right)=\underset{\gamma \in \Pi (p_{real},p_{s(t)})}{\inf}E_{(x,y)∼\gamma }\left[∥x-y∥\right]$$

In this context, $\Pi (p_{real},p_{s(t)})$ represents the set of all joint distributions γ(x,y) whose marginal distributions are $p_{real}$ and $p_{s(t)}$, respectively. Martin et al. interpret this as the “mass” transported from *x* to *y* to transform the distribution $p_{real}$ into $p_{s(t)}$, with Wasserstein distance reflecting the optimal transportation cost. The challenge lies in finding the infimum (the greatest lower bound) of the set or function described in (3), which is noted to be very difficult to compute or solve. To address this, weight clipping is employed to constrain the weights of the discriminator neural network within a specific range, preventing excessive weight changes that could lead to model instability. In WGAN, the discriminator is referred to as the critic. Martin et al. used a default clip value of 0.01 during image generation, while this study explored clip values ranging from 0.1 to 0.001, ultimately determining that a value between 0.05 and 0.1 yielded the best model performance.

### 2.4. ViT for fMRI (VTFF)

For the core neural networks of the generator and discriminator, the VTFF model was selected [32]. Unlike traditional multilayer perceptrons and other deep learning techniques, this model is designed to adapt the Vision Transformer (ViT) model [21] for fMRI data pattern recognition. The ViT architecture is based on Transformers and incorporates an encoder structure. In the patching embedding layer of the input images, a class token is added, and attention is calculated between nodes through successive Transformer Blocks (4) and (5). In the output layer, the features aggregated from the class token across different layers are combined using the softmax function to achieve multi-class discrimination.

In contrast to 2D image recognition, fMRI data is 4D, encompassing both a temporal axis and three-dimensional brain imaging. Therefore, when applying transfer learning to the ViT within the VTFF model, the process first flattens the data from four-dimensional space to two-dimensional and then to one-dimensional space along the temporal axis. This generates a collection of patches representing the entire brain’s fMRI signals, defined as a TS-wise strategy. The whole-brain fMRI signals can be allocated to N brain regions (number of brain regions, NB) based on brain region partitioning methods, resulting in whole-brain fMRI signals of size (Timepoints×NB). Consistent with the scale of the previously simulated fMRI signals, the input shape for the VTFF layer is defined as (32, 100, 360), representing a batch size of 32, a length of 100 time points for fMRI, and 360 multi-modal cortical regions. The partitioning method will be described in detail in the preprocessing of the real fMRI data.

(4) $$Attention\left(Q,K,V\right)=softmax\left(\frac{QK^{T}}{\sqrt{d_{k}}}\right)V$$

Equations (4) and (5), proposed by Vaswani et al. [8], describe the computation of multi-head attention within the Transformer Block. In this context, Q, K, and V refer to the Query, Key, and Value vectors, respectively, while $d_{k}$ represents the dimensionality of the embedding vectors, which is typically set to 512 in both Transformer and ViT models. In this study, however, a value of 360 is employed to correspond with the number of brain regions. The term $W^{O}$ signifies the learnable weight information used for computing the concatenated multi-head attention, and HPTN represents the number of heads in each Transformer Block. By default, this is set to 12, in alignment with the ViT-Base model.

(5) $$MA\left(Q,K,V\right)=Concat\left(head_{1},\ldots ,head_{HPTN}\right)W^{O}$$

In the previous section, Figure 1A utilized two variations of the VTFF model (in the generator and critic), while Figure 1C presents the basic version of VTFF (in the classifier). The core component of both models employs a 12-layer Transformer Block structure (in blue), with the primary differences lying in the input data types and the output network architectures.

For the generator, the input layer receives fMRI simulation signals. After being processed through the stacked Transformer Blocks, the final layer directly outputs the fake fMRI signals, maintaining the same shape as the input layer, which is (32, 100, 360).

For the critic, the input layer receives either fake fMRI or real fMRI data. It is tasked with performing binary classification to determine the authenticity of the input data. To achieve this, the concept of embedding the class token from the ViT model is employed. In the patch embedding process, a class token of size (1, 360) is concatenated with the input layer of size (100, 360). This concatenated data is then stacked in the VTFF network as a Transformer Block with a shape of (32, 101, 360). After the Transformer Matching Layer (in orange), the class token undergoes a linear transformation and is mapped to a probability value in the range of [0, 1] using a sigmoid function. This layer primarily evaluates the feature differences between real and fake data, serving as a GAN network enhancement technique, which will be elaborated on further.

For the classifier, the input data consists of a mixture of real fMRI and the generated fMRI, which has achieved a certain balance through multiple rounds of the “generation-discrimination” adversarial model. The network structure of the classifier remains consistent with the basic version of VTFF, with the input layer size being (32, 101, 360), including the class token. The data is processed through 12 layers of stacked Transformer Blocks, and the softMax layer is employed for multi-class classification of the test data.

### 2.5. WGTMM: WGAN with Transformer Matching

In WGAN, the generator attempts to produce realistic data by maximizing its ability to deceive the critic. However, this adversarial mechanism can sometimes lead to the generator learning to produce only a limited variety of samples. This issue is particularly pronounced with fMRI data, where visually observing sample differences is nearly impossible, potentially resulting in a lack of data diversity and causing GAN mode collapse. To address this, feature matching techniques [20,33] are employed, using the output of a specific intermediate layer of the discriminator as a feature representation of the samples, thereby measuring the feature differences between real and fake samples, as shown in (6). Here, TB denotes the last layer of the Transformer Block, serving as the feature matching (FM) layer (depicted in orange in Figure 1 within the Critic of the VTFF model), E represents the expected value, $x_{real}$ indicates the real data, $p_{real}$ is the distribution of the real data, and *f* is the feature representation of the input data at that layer. In practice, the degree of feature matching difference can guide the tuning direction of the generator’s loss function within the WGAN network. Thus, during the iterative process, the updated generator loss is minimized as represented in (7). Here, λ is the feature matching coefficient used to assess the influence of WGAN loss and the feature matching across various degrees of the Transformer. Given that the critic is designed to output higher values for real samples and lower values for generated samples, the generator’s objective is to maximize the expected critic score of the generated samples. To formulate this as a standard minimization problem for gradient descent, a negative sign is introduced, resulting in the expression $-E_{s\left(t\right)∼p_{s(t)}}\left[D(G\left(s\left(t\right);\theta_{G}\right))\right]$. Consequently, as the critic successfully learns to widen the scoring gap by assigning larger positive values to real data and lower values to generated data, the overall adversarial loss curve naturally decreases into negative territory. Unlike the strictly positive cross-entropy loss in traditional GANs, this negative trajectory in WGAN is an expected behavior that mathematically indicates stable optimization and proper convergence.

(6) $$\begin{array}{c} TB_{FM}=∥E_{r_{real}∼p_{real}}\left[f(x_{real})\right]-\\ E_{s\left(t\right)∼p_{s(t)}}\left[f(G\left(s\left(t\right);\theta_{G}\right)))\right]{∥}_{2}^{2} \end{array}$$

(7) $$Loss=-E_{s\left(t\right)∼p_{s(t)}}\left[D(G\left(s\left(t\right);\theta_{G}\right))\right]+\lambda TB_{FM}$$

### 2.6. In Vivo fMRI Acquisition and Data Preprocessing

The data utilized in this study is identical to that used in previous research [34,35,36], sourced from the ADNI dataset, as detailed in Table 1. The fMRI data consist of time series with a repetition time (TR) of approximately 2–3 s depending on the ADNI acquisition protocol. The advantage of using the same publicly available dataset lies in the ability to thoroughly compare the performance improvements offered by different network models. However, the downside is evident, as the generalizability of the results remains to be validated. Therefore, efforts are made to collect as much fMRI data as possible to mitigate the effects of model overfitting. Concurrently, a WGAN network featuring Transformer-based feature matching is proposed, training models separately for patients with varying degrees of cognitive impairment. This process generates a total of 320 × 4 sets of data, each annotated with the corresponding labels.

The previous section mentioned that the input layer shapes for the generator, critic, and classifier are all (32, 100, 360), where 32 represents the batch size, 100 denotes the number of time points in the fMRI data, and 360 corresponds to the number of brain regions. For the simulated fMRI data, 360 groups of pink noise can be randomly generated, while real fMRI data requires data preprocessing. The preprocessing workflow typically includes slice timing correction, motion correction, artifact detection, co-registration, and normalization, aiming to align functional magnetic resonance images with structural magnetic resonance images and achieve coordinate space transformation. The HCP MMP cortical parcellation method [25] proposed by the University of Washington divides the human cerebral cortex into 180 regions in each hemisphere based on four modalities: architecture, function, connectivity, and topology. Although this method is based on data from the HCP protocol, the earlier data collection standards of the ADNI database did not meet the high-quality requirements set by HCP. Therefore, this study employs the JHCPMMP research method, integrating tools such as FreeSurfer, fMRIPrep, and CIFTIFY [37,38,39], to achieve fine-grained multimodal HCP MMP parcellation of the non-HCP ADNI data.

### 2.7. Evaluation for Mode Collapse in fMRI-Related GANs

To assess the diversity of fMRI data generated by various GAN networks and to reduce the occurrence of mode collapse in the generator, this study employs the Kullback-Leibler (KL) [40] divergence calculation method based on kernel density estimation to measure the differences between generated samples and the empirical fMRI samples within the observed cohort. Equation (8) presents the calculation method for the kernel density estimation function [41], which is used to estimate the probability density function $\hat{p}$ of the random variable (i.e., the fMRI time series). A time series within a specific brain region is defined as $T(T_{1},T_{2},T_{3},\ldots ,T_{n})$, with a length of n(n=100) time points. Here, *K* represents the Gaussian kernel function, and *h* is the bandwidth parameter, following the Silverman method [42]. By substituting the fake fMRI generated by the generator and the real fMRI time series from the ADNI database into (8), the discrete KL values $D_{KL}(\hat{fake}\left(T\right)||\hat{real(T)})$ for each corresponding brain region can be calculated (as shown in (9), where $\hat{fake}$ and $\hat{real}$ denote the density estimates for the fake and real fMRI data, respectively. Equation (10) calculates the average KL value for each brain region, which serves as the KL divergence between fake samples and real samples. Ultimately, M×NKL values were calculated across four sample groups to represent the pairwise differences between the generated data (M samples) and real data (N samples), thereby evaluating the degree of mode collapse in the GAN networks. A lower KL value indicates higher distributional consistency within the observed cohort, while a higher KL value signifies a larger discrepancy between the generated data and the real situation.

(8) $$\hat{p}\left(T\right)=\frac{1}{nh}\sum_{i=1}^{n}K\left(\frac{T-T_{i}}{h}\right)$$

(9) $$D_{KL}(\hat{fake}\left(T\right)||\hat{real(T)})=\sum_{x}\hat{fake}\left(T\right)log\left(\frac{\hat{fake}\left(T\right)}{\hat{real}\left(T\right)}\right)$$

(10) $$\bar{KL}=\frac{1}{ROIs}\sum_{j\in ROIs}^{ROIs}D_{KL,j}$$

### 2.8. Classification Evaluation

The evaluation is established on an 80%:20% random split of the ADNI dataset, yielding a held-out real test set of 112 HC, 119 EMCI, 83 LMCI, and 46 AD subjects that serves as the exclusive benchmark for all classification metrics. Initially, a baseline VTFF classifier is trained solely on the real ADNI training portion. To justify the efficacy of the proposed generation method prior to downstream enhancement, this baseline model is applied directly to the synthetic fMRI samples generated by the WGTMM model. These generated samples are leveraged strictly for cross-distribution testing during this preliminary evaluation, ensuring that the initial VTFF training phase remains entirely uncontaminated by synthetic data.

To fully exploit the generative framework for performance optimization, synthetic fMRI samples derived respectively from GAN, WGAN, and WGTMM are integrated into the real ADNI training pool, constructing distinct augmented configurations. We then train separate VTFF classifiers on each augmented dataset to investigate how different generative profiles influence model learning.

The ultimate generalization capacity of these augmented models is validated by re-evaluating them against the identical, baseline real ADNI test set. By deriving the final diagnostic labels from the highest posterior probabilities of the softmax outputs, the resulting confusion matrices allow for a direct performance comparison, verifying whether synthetic data augmentation successfully translates into tangible gains on real-world clinical data. Lastly, an adversarial stability evaluation via label perturbation is introduced to verify whether the model captures genuine pathophysiological features rather than memorizing label couplings.

## 3. Results

**Figure 2:**
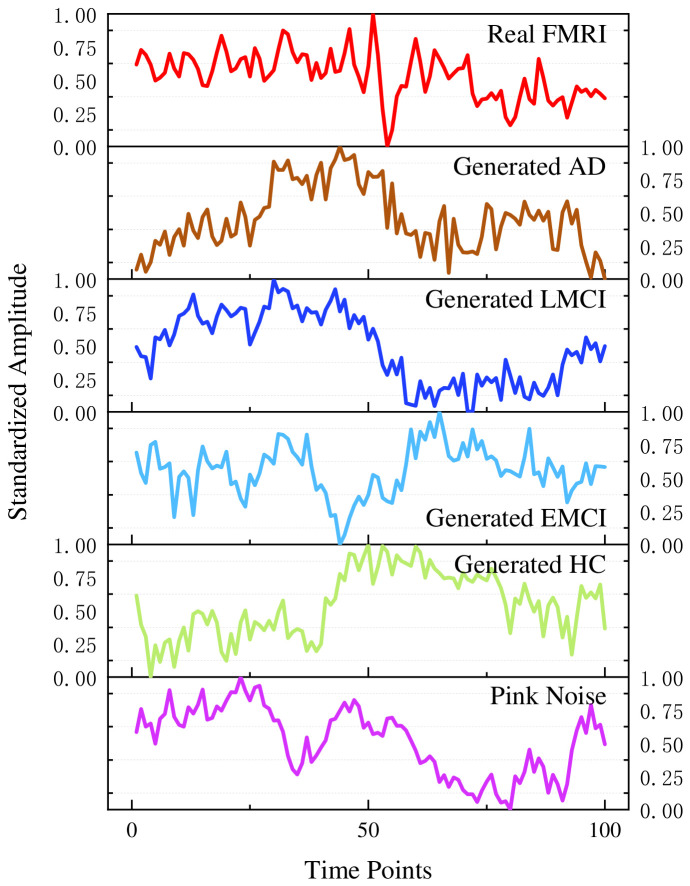
Pink noise and generated fMRI signals, along with real fMRI data downloaded from ADNI in this study.

Figure 2 presents the pink noise (Pink), real fMRI signals (Red), and fMRI signals generated by the WGTMM model corresponding to various stages of cognitive impairment (light blue for early MCI (EMCI), dark blue for late MCI (LMCI), and brown for AD), as well as healthy control fMRI signals (Green). All signals have been min-max normalized to a unified scale of [0, 1] on the Y axis to eliminate any visual illusions regarding amplitude differences that might arise from unscaled auto-plotting. As shown in the figure, pink noise exhibits a higher concentration of low-frequency components, which are noticeably reduced in the generated fMRI signals. This indicates that the GAN-generated signals differ from random noise, suggesting that they are not purely random but exhibit structured and characteristic time-series patterns. It is worth noting that the generated signals exhibit more high-frequency fluctuations compared to the real fMRI signals. This discrepancy stems from the inherent characteristics of the simulation process; while real BOLD signals undergo a natural low-pass filtering effect governed by the physiological HRF that smooths out rapid fluctuations, the generated data retains these mathematical high-frequency noise components introduced during the simulation. However, despite these fine-grained noise differences, visually distinguishing the core patterns of the generated signals from real fMRI signals, especially across different clinical categories, remains challenging. Further statistical analysis and pattern recognition methods are needed to identify the essential differences between the generated and real signals.

**Figure 3:**
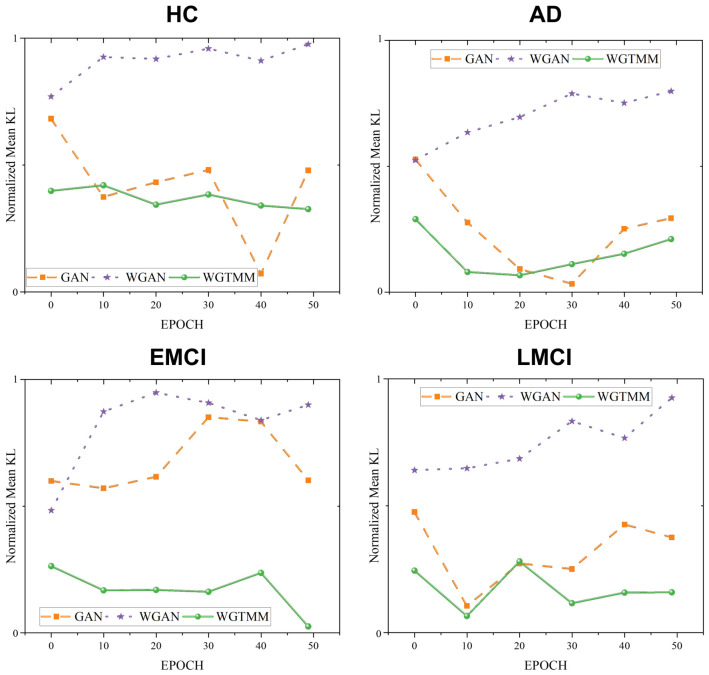
Average KL divergence levels of the four datasets generated by the GAN, WGAN, and WGTMM models.

Figure 3 illustrates the KL divergence distribution of fMRI data generated by the GAN, WGAN, and WGTMM networks. During the training process, this study generated fMRI data every 10 epochs, producing 320 fake fMRI samples for each of the four groups: HC, EMCI, LMCI, and AD. The KL divergence between these generated samples and real fMRI data was then calculated. The x-axis represents the epochs, while the y-axis shows the normalized average KL values. The orange, purple, and green curves correspond to the GAN, WGAN, and WGTMM models, respectively.

From the figure, it is evident that the KL values of fMRI generated by the WGTMM model are consistently the lowest across all four categories, indicating the closest alignment with the distribution of real fMRI data. In contrast, the WGAN network exhibits relatively high KL values across all classifications. The GAN network demonstrates a gradual convergence towards the real data distribution as the model iterations increase, yet its overall KL level is still inferior to that of the WGTMM model. The average KL values provide an assessment of the data quality generated by different GAN networks and help identify potential mode collapse phenomena that might otherwise be overlooked. However, to fully evaluate the effectiveness of the model training, further analysis using confusion matrices will be necessary.

**Figure 4:**
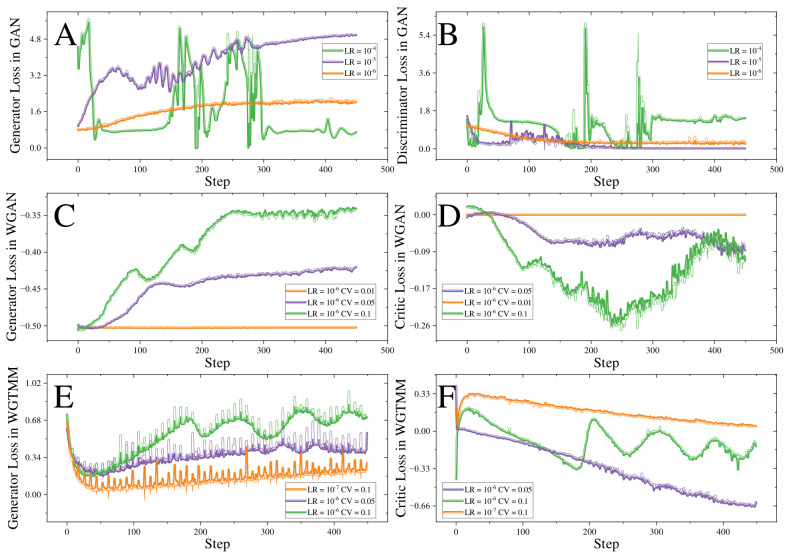
Loss conditions of the generator and discriminator in different GAN networks. The left side (**A**,**C**,**E**) represents the generator, while the right side (**B**,**D**,**F**) represents the discriminator. (**A**,**B**) corresponds to the GAN network, (**C**,**D**) corresponds to the WGAN network, and (**E**,**F**) corresponds to the WGTMM network. The horizontal axis represents training steps, and the vertical axis represents loss values. Due to the different definitions of loss functions across models, no horizontal comparison was made; instead, ablation experiments were conducted under different network parameters.

Figure 4 displays the loss dynamics of the generator and discriminator across three models, along with the results of ablation experiments corresponding to different network parameters. The left column represents the generator, while the right column represents the discriminator. In the GAN network (Figure 4A,B), various learning rates ($10^{-4}$, $10^{-5}$, and $10^{-6}$) were tested. It is observed that when the learning rate is set to $10^{-6}$, both the generator and discriminator show minimal changes, exhibiting a slow, unidirectional trend. The generator’s loss gradually increases, while the discriminator loss experiences a slight decline, indicating that the excessively low learning rate leads to slow convergence of the model, resulting in generated fMRI data that is nearly indistinguishable from noise. As the learning rate is increased to $10^{-5}$, the generator experiences a significant loss, continuing to show a general upward trend, while the discriminator’s loss remains relatively low and stable. This suggests that the generator finds it increasingly difficult to deceive the discriminator, which has not learned any useful weight information and can easily identify the fake data. When the learning rate is raised to $10^{-4}$, a competitive dynamic between the generator and discriminator emerges, reflected in the loss values. The generator’s initial high loss sharply decreases and fluctuates during subsequent training iterations. Overall, the generator’s ability to produce fake fMRI data remains relatively stable, without significant improvements in model generation or discrimination capabilities despite the increase in iteration count.

Figure 4C,D presents the loss dynamics for the generator and discriminator (critic) within the WGAN network, where the impact of different clip values (CV = 0.1, 0.05, and 0.01) is evaluated. During our hyperparameter tuning, several learning rates (ranging from $10^{-4}$ to $10^{-6}$) were initially tested. However, the network was highly sensitive to this parameter, and learning rates higher than $10^{-6}$ resulted in complete failure to converge (showing almost no learning capability). Therefore, to provide a meaningful and clear comparison of the optimization dynamics, we fixed the learning rate at the optimal value of $10^{-6}$ across these plots, while explicitly demonstrating how different clip values affect the training stability. When the CV is set to 0.01, the model exhibits almost no learning capability. However, as the CV is gradually increased, both the generator and discriminator losses decrease, indicating a competitive dynamic. Initially, the discriminator’s loss declines while the generator’s loss increases, suggesting that the generator is producing low-quality fMRI data. After approximately 100 training steps, the fMRI generated by the generator begins to pose increasing challenges for the discriminator, reflected in a rise in the discriminator’s loss, and this adversarial process continues throughout the training. Overall, as training progresses, the adversarial game stabilizes, with both curves flattening out to a steady state, indicating that the model has reached proper convergence under optimized hyperparameter constraints.

Figure 4E,F illustrates the training dynamics of the proposed WGTMM model. It is evident that when the LR is set to $10^{-6}$ and the CV is 0.1, the adversarial interactions between the generator and discriminator are quite pronounced. Initially, the generator produces fMRI data that fails to deceive the discriminator, resulting in a rapid increase in loss. However, within a few training steps, a decline in loss occurs, indicating an increase in the discriminator’s challenge in making accurate judgments.

This adversarial process continues throughout the entire training cycle, demonstrating a consistent improvement in the quality of the generated fMRI data, making it increasingly difficult for the discriminator to distinguish between real and fake fMRI characteristics.

**Figure 5:**
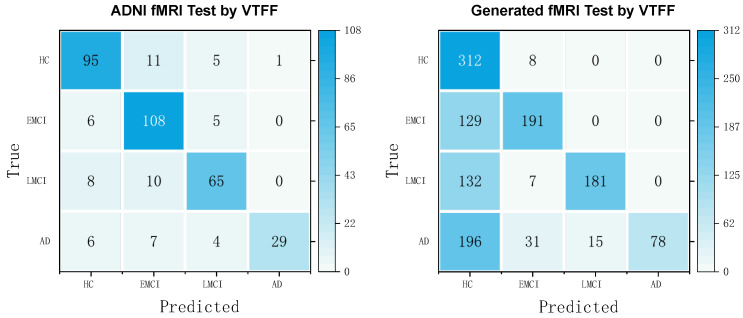
Cross-distribution evaluation of the baseline VTFF classifier trained exclusively on the real ADNI training partition. The left matrix illustrates performance on the 20% held-out real ADNI test subset (comprising 112 HC, 119 EMCI, 83 LMCI, and 46 AD subjects), while the right matrix evaluates the model against the synthetic fMRI samples generated by WGTMM, featuring a balanced set of 320 independent samples for each of the four diagnostic categories to validate generation fidelity.

The fake fMRI generated by the previous three methods was used to train a classification model for cognitive impairment populations, aiming to assess whether generative techniques could enhance model recognition capabilities. Initially, the VTFF model trained on the ADNI dataset (as shown in Figure 1C) was applied to identify both the ADNI dataset and the fake fMRI generated by WGTMM. The multi-class confusion matrix is depicted in Figure 5.

The results indicate that the VTFF model achieves classification accuracies of 63% for AD, 90.8% for EMCI, 78.3% for LMCI, and 84.8% for HC on the ADNI dataset. In contrast, the performance on the WGTMM-generated fMRI is relatively poor, with accuracies of only 24.4% for AD, 59.7% for EMCI, 56.6% for LMCI, and 97.5% for HC.

**Figure 6:**
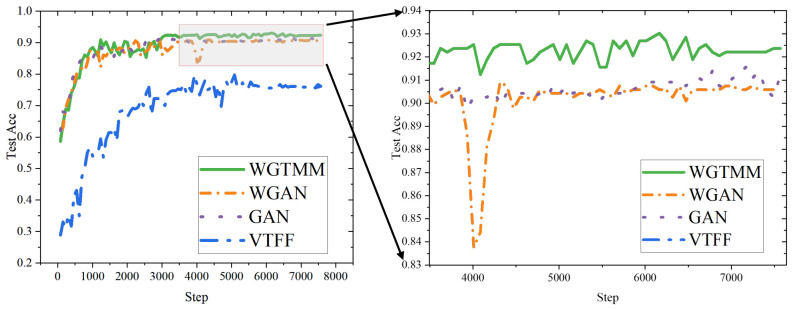
Test accuracy curves as a function of training steps for the VTFF model trained and evaluated on mixed datasets (real ADNI combined respectively with GAN, WGAN, and WGTMM generated fMRI data).

**Figure 7:**
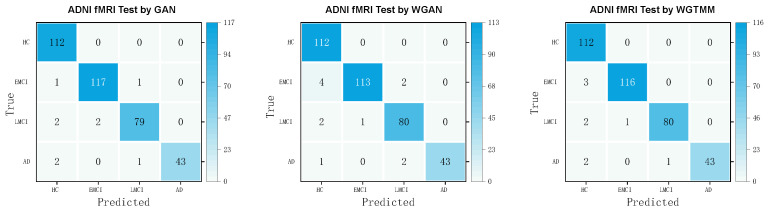
Final confusion matrices obtained on the held-out real ADNI test set for VTFF classifiers trained under GAN, WGAN, and WGTMM data augmentation configurations.

After mixing the generated fMRI with the ADNI data to form an enhanced mixed dataset, the model’s performance was retested, as shown in Figure 6. The blue curve represents the performance of the original VTFF model, while the green, purple, and orange curves correspond to the testing accuracy changes when fMRI generated by WGTMM, WGAN, and GAN networks is mixed with ADNI data. All data generation methods significantly improve VTFF performance over training on ADNI data alone, with WGTMM performing best. In Figure 7, the three enhanced models were applied to the final test on the ADNI dataset to evaluate the effectiveness of adversarial augmentation techniques in recognizing fMRI in real-world scenarios. The confusion matrix results and the effects of different data augmentation strategies are presented in Table 2.

**Figure 8:**
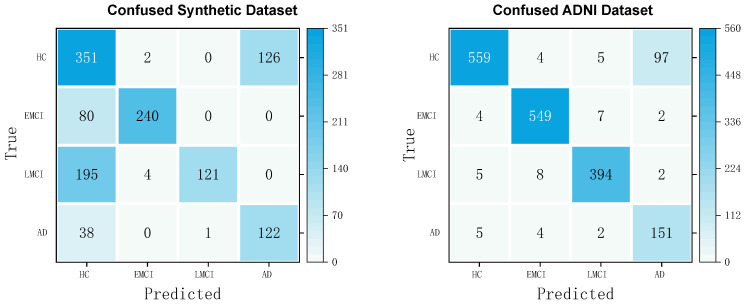
Confusion matrices of the VTFF classifier under the adversarial label-perturbation stress test. Despite deliberately mislabeling half of the synthetic AD samples (160 out of 320) and an additional 100 real AD samples as HC during evaluation, the classifier successfully resisted the label corruption, robustly identifying these perturbed samples as AD based on their intrinsic fMRI pathophysiological features.

Meanwhile, we conducted additional experiments to assess whether the model’s recognition ability has genuinely improved, rather than being influenced by label coupling or other training factors that lead to high classification levels. For example, we introduced label noise by systematically perturbing the true labels during evaluation. Specifically, for the synthetic dataset, we mislabeled half of the generated AD samples (160 out of 320 samples) as HC. Concurrently, for the real ADNI dataset, 100 real AD samples were also mislabeled as HC. Figure 8 shows the four-class confusion matrix after label confusion, with the left side representing the confused fabricated fMRI data and the right side representing the confused ADNI data. It can be observed that the label confusion has no impact on the model’s detection results, as it still correctly identifies the fMRI data. In the fabricated fMRI dataset, 126 HC samples were predicted as AD, while in the ADNI dataset, 97 HC individuals were identified as AD patients. The model’s average accuracy still exceeds 95% (1750/1798), demonstrating the stability of the model’s classification performance.

## 4. Discussion

This study explores the application of adversarial generation techniques in fMRI data augmentation, utilizing a GAN framework based on the VTFF model. We trained three network architectures, GAN, WGAN, and WGTMM, to generate fabricated fMRI data representing varying degrees of cognitive impairment. These generated data were then mixed with real ADNI data to enhance the capability of recognizing cognitive impairments. Through this data augmentation strategy, the research aims to improve the robustness and accuracy of classification models, thereby facilitating early diagnosis of cognitive impairments. Additionally, the study evaluates the performance of different network architectures in terms of data quality and diversity, aiming to identify the optimal generation model.

### 4.1. Adversarial Generation Techniques Enhance Recognition Abilities of Cognitive Impairment Models

Compared to traditional methods that solely rely on real neuroimaging datasets for training models, the GAN-based fMRI data augmentation technique significantly enhances the performance of classification models for various degrees of cognitive impairment. This is clearly observed in Figure 6. Currently, machine learning algorithms, such as decision trees and SVM, have achieved high levels of performance in addressing binary classification problems for cognitive impairment, with an average accuracy exceeding 90%. However, in the context of multi-class problems, the field of cognitive neuroscience still faces numerous challenges, particularly since we cannot restrict the model to only process two classes of samples in real-world applications. To address this, the VTFF method has been proposed, which migrates the recently successful Transformer and ViT models to the processing of fMRI data, achieving nearly 80% classification capability for four classes. This achievement provides a new approach to tackling multi-class problems. Building on VTFF, the proposed WGTMM method enhances fMRI data, successfully increasing the classification level by 10 percentage points. This significant progress not only improves the model’s ability to recognize cognitive impairments but also provides crucial support for further exploring the commonalities between real and fabricated fMRI data. By analyzing the augmented data, we can reveal the underlying features and patterns within neuroimaging data, which will help in better understanding the variations in brain activity across different cognitive states. To the best of our knowledge, this is the first study to simulate fMRI BOLD signals at a fine-grained level, distinguishing between EMCI and LMCI stages of cognitive impairment. In contrast, previous studies [43,44,45] typically report results using a single, unified MCI category. To enable fair comparison, we retained the separate EMCI and LMCI categories in our table but duplicated the same MCI results from prior work across both columns, ensuring alignment of confusion matrix metrics in Table 2. Compared to these latent space-based methods, our approach directly maps the output layer of the attention network to synthetic fMRI BOLD time series. This better preserves the temporal characteristics of fMRI signals and captures common patterns of brain abnormalities across different degrees of cognitive impairment.

### 4.2. Transformer-Based Feature Matching Enhances Fabricated fMRI Data Quality

Combining Figure 3 and Figure 4, it is evident that the feature matching technique based on Transformer plays a significant role in adversarially generated fMRI data, effectively enhancing the quality of fabricated fMRI data. Although the KL values for the original GAN network were lower than those of the WGTMM model in the HC and AD groups in Figure 3, the average KL levels and their trends with increasing epochs indicate that the fMRI data generated by the WGTMM network are closer to the real fMRI samples from the ADNI dataset, with smaller KL values compared to the other two methods. Interestingly, the fMRI data generated by the WGAN network, defined by Wasserstein distance, differ significantly from the distribution of real fMRI data, suggesting a severe mode collapse that hinders the iterative generation of high-quality fMRI data. However, considering the classification results in Figure 6 and Figure 7, and Table 2, the WGAN-based network still achieves high classification accuracy. Nonetheless, its high KL divergence suggests that the generated data may lack sufficient diversity—likely a result of mode collapse producing many similar samples. Therefore, for studies aiming to generate fMRI data to enhance cognitive model training, classification performance alone may be insufficient to evaluate the quality of generated data. Additional metrics such as KL divergence are essential for assessing data diversity and realism. The loss curve changes in Figure 4 also reflect this point. For the GAN network, although both the generator and discriminator experience significant fluctuations in performance during their adversarial game, indicating ongoing attempts to deceive each other, the overall loss level does not show significant improvement. The WGAN network exhibits a monotonous trend with local adversarial features. In the WGTMM model, the oscillation of loss values for the generator and critic corresponds with the high classification performance in Figure 6, suggesting that the enhancement of model performance is accompanied by an adversarial game process where both parties improve gradually over time.

### 4.3. WGTMM Method Reveals Common Features Between Real and Fabricated fMRI Data

**Figure 9:**
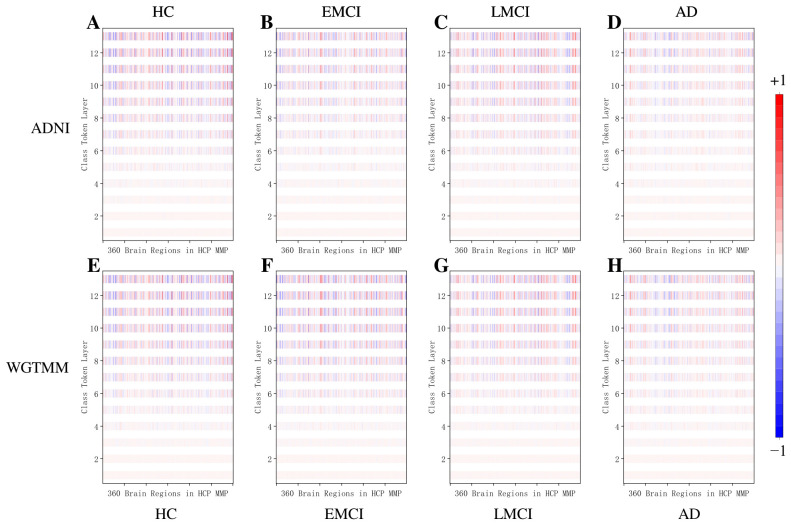
Layer-wise changes of class tokens in the VTFF model. (**A**–**D**) represent the HC, EMCI, LMCI, and AD in the ADNI dataset, while (**E**–**H**) are fMRI data fabricated by WGTMM. The x-axis corresponds to the 360 multimodal HCP MMP brain regions, and the y-axis represents the values of each layer’s class tokens. Each heatmap is composed of 360 × 13, with the first 12 layers being Transformer Block layers and the final layer being the feature matching layer, consistent with the network structures defined in Figure 1.

**Figure 10:**
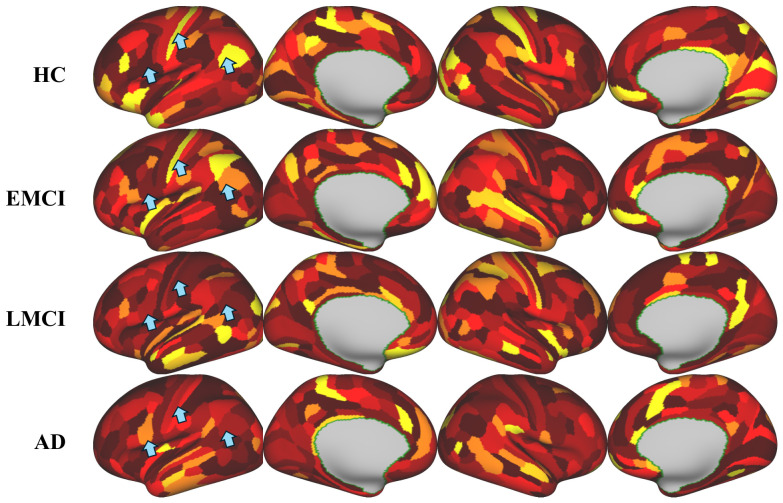
Mapping of the feature matching layer across brain regions in samples with varying degrees of cognitive impairment. The sequential red-orange-yellow colormap encodes the relative magnitude of model weights.

Direct observation of fMRI data is quite challenging. Previous studies have primarily relied on statistical analysis, topological analysis, or machine learning methods to investigate the patterns of brain variations in patients with varying degrees of cognitive impairment. In this paper, we utilize an adversarially trained WGTMM model to generate sufficiently realistic fMRI data, allowing us to reveal the roles of different brain regions in cognitive impairment identification models by comparing the common features between real and fabricated fMRI data. In the ViT-like model, the layer-wise aggregated class tokens serve as the most important basis for model decisions. Consequently, Figure 9 illustrates the layer-wise changes of class tokens in the VTFF model used in this study. The x-axis represents the 360 HCP MMP brain regions, while the y-axis shows the values of the 13 layers of class tokens (comprising 12 layers of Transformer Blocks and the final feature matching layer). We observe that the VTFF class tokens trained on real ADNI data and those generated using the WGTMM model display specific patterns of change across layers. Although the variations in class tokens are diverse and converge relatively slowly in the initial Transformer Blocks, a stable pattern generally emerges after layers 5–6 and continues through to the feature matching layer. The class tokens for three different degrees of cognitive impairment and the healthy control group exhibit markedly different patterns in the final layer, yet the results from the ADNI dataset and WGTMM data are very close. Furthermore, by mapping the feature matching layer to the HCP MMP cortical surface (as shown in Figure 10), we can visually observe the differing expressions of various brain regions at the classification layer. In several widely recognized cognitive-related brain regions (indicated by arrows, such as Rostral Area 6 near Broca’s area, the primary sensory cortex, and the PFm cortex near Wernicke’s area), we also observed a monotonic change in weight expression that gradually decreases or increases. In-depth research in this area has the potential to provide effective biomarkers for cognitive impairment.

### 4.4. Limitations

A key limitation of this study is that all experiments were conducted using a single dataset, the ADNI database. Relying on one dataset may introduce biases in pattern recognition and affect the generalizability of the data generation quality. Although ADNI is a large-scale, multi-center international neuroimaging dataset widely recognized for its variability due to standardized imaging protocols combined with differences in scanning equipment and repeated measurements over time, validating the model on additional independent datasets would further enhance its robustness and generalizability.

## 5. Conclusions

In this study, we proposed the Wasserstein GAN with Transformer Feature Matching (WGTMM) method, which utilizes the VTFF model as the core generator and discriminator of the GAN network. By integrating the Wasserstein loss function with Transformer Feature Matching techniques, we ensure that the pink noise data generated during iterations closely aligns with the feature layer outputs of real fMRI data, thereby facilitating the generation of synthetic fMRI data representing varying degrees of cognitive impairment. Through the analysis of KL divergence and model loss, we compared our WGTMM model with other adversarial generation networks such as GAN and WGAN. Our observations indicated that the mode collapse phenomenon was least pronounced in the WGTMM model, and the similarity between the generated fMRI data and the authentic ADNI fMRI data was notably high. Given the noise present in BOLD signals resulting from differences in acquisition devices and various imaging factors, the synthetic fMRI data reflecting different levels of cognitive impairment can effectively enhance model classification performance. This advancement is beneficial for studying the common characteristics shared between real and synthetic fMRI data, which could help uncover the underlying patterns of brain function. Additionally, we observed a monotonic variation in weight expressions, which either gradually decreased or increased in brain regions such as the Rostral Area 6 near Broca’s area, the primary sensory cortex, and the PFm area near Wernicke’s area. Further investigation in this area holds promise for identifying effective biomarkers for cognitive impairment.

## Figures and Tables

**Table 1:** Statistical Information of Sample Data and Training Generated fMRI Data.

	HC	EMCI	LMCI	AD
Real fMRI Samples	567	562	409	260
Male/Female	271/296	316/246	210/199	145/115
Age	72.18 ± 11.69	70.32 ± 8.39	68.97 ± 13.47	73.45 ± 10.96
Education Years	16.12 ± 3.21	16.14 ± 2.89	16.52 ± 3.64	15.61 ± 2.96
CDR	0.034 ± 0.14	0.47 ± 0.19	0.55 ± 0.34	0.89 ± 0.41
MMSE	28.94 ± 1.35	28.12 ± 1.83	26.66 ± 3.40	21.6 ± 3.74
NPI	1.54 ± 4.72	4.22 ± 7.18	5.18 ± 7.68	7.72 ± 9.56
GDS	0.78 ± 1.26	1.94 ± 1.89	1.84 ± 2.06	1.72 ± 1.72
FAQ	0.28 ± 1.37	2.65 ± 4.22	5.13 ± 6.81	14.84 ± 7.71
ADAS	8.70 ± 4.95	12.45 ± 6.72	18.90 ± 10.79	32.97 ± 11.15
Generated fMRI Samples	320	320	320	320

**Table 2:** Final classification performance on the held-out real ADNI test set under different generative data augmentation configurations.

Model	Average	HC			EMCI			LMCI			AD		
	Acc	P	S	F1	P	S	F1	P	S	F1	P	S	F1
VTFF on fake only	0.60	0.49	0.98	0.65	0.81	0.60	0.69	0.92	0.96	0.94	1.00	0.24	0.39
Basu et al. [43]	0.76	0.83	0.81	0.82	0.66	0.68	0.67	0.66	0.68	0.67	0.83	0.81	0.82
Hazarika et al. [44]	0.80	0.83	0.88	0.85	0.75	0.71	0.73	0.75	0.71	0.73	0.83	0.88	0.85
Gao et al. [45]	0.85	0.88	0.90	0.89	0.79	0.78	0.79	0.79	0.78	0.79	0.88	0.90	0.89
Wang et al. [32]	0.83	0.83	0.85	0.84	0.79	0.91	0.85	0.88	0.78	0.83	0.97	0.63	0.76
VTFF on WGAN mix	0.97	0.94	1.00	0.97	0.98	0.95	0.97	0.95	0.96	0.96	1.00	0.93	0.97
VTFF on WGTMM mix	0.98	0.94	1.00	0.97	0.98	0.97	0.98	0.96	0.96	0.96	1.00	0.93	0.97

Acc: Accuracy, P: Precision, S: Sensitivity, F1: F1-score. Results obtained from Gao et al. [45] (Table IV) by averaging scores in AD vs. NC and MCI vs. NC.

## Data Availability

The original contributions presented in this study are included in the article. Further inquiries can be directed to the corresponding author.
